# Corn leaf disease diagnosis: enhancing accuracy with resnet152 and grad-cam for explainable AI

**DOI:** 10.1186/s12870-025-06386-0

**Published:** 2025-04-07

**Authors:** Kirubasri Gopalan, Saravanan Srinivasan, Pragya Agarwal, Manu Singh, Sandeep Kumar Mathivanan, Usha Moorthy

**Affiliations:** 1 Department of Computer Science and Engineering, Sona College of Technology, Salem, Tamil Nadu India; 2 Department of Computer Science and Engineering, Vel Tech Rangarajan Dr. Sagunthala R&D Institute of Science and Technology, Chennai, India; 3 School of Computer Science and Engineering, Galgotias University, Greater Noida, 203201 India; 4 Department of Information Technology, Manipal Institute of Technology Bengaluru, Manipal Academy of Higher Education, Manipal, 576104 Karnataka India

**Keywords:** Corn leaf disease, Deep learning, ResNet152, Grad-CAM, Explainable AI

## Abstract

**Objective:**

The agricultural sector is important in the supply of food globally as well as in enhancing the economy especially in developing countries where it forms the backbone of the economy. Corn can be classified as such crops important to the world’s food system. Unfortunately, corn crop is vulnerable to a lot of diseases and this may result into heavy losses and disruption in the food supply system. Hence, it is important to detect and classify these diseases accurately and promptly to limit losses and achieve the highest possible productivity. This study intends to solve these problems by constructing a trustworthy and interpretable model based on deep learning approaches focused on accurate identification of corn leaf disease.

**Material:**

In this study, the cumulative dataset comprises 4188 images which are further divided into four classes of corn leaf disease, with 1146 images of blight leaf, 1306 images of common rust, 574 images of gray spot and 1162 images of healthy leaves. In order to train and validate the model 70% of data was used for training while 30% was used for testing. This division was appropriate because it allowed enough data to be used during model training and also enough for model evaluation on new data.

**Methods:**

The research employs ResNet152, a well-known deep leaning structure in image classification, because uses residual connections that improve the training of deep networks. Furthermore, Grad-CAM (Gradient-weighted Class Activation Mapping) is employed to improve the explainability of the model. Grad-CAM produces human interpretable visual images in the form of heatmaps and it indicates the areas of corn leaves that have had the greatest impact in the model, which is fairly useful in understanding the model. The model processes and predicts the corn leaves into four classes: healthy (H), blight (B), gray spot (GS) and common rust (CR), with precision and explainability.

**Results:**

The results of training the ResNet152 model were remarkable as it registered a 99.95% accuracy during training as well as 98.34% during testing. Also, applying Grad-CAM for interpretability purposes proved to be useful as it created heatmaps that indicated the most important parts of the leaf images for making the model predictions. This added to the understanding of the model and its predictions, which was especially important for users such as farmers who required accurate diagnoses of diseases.

**Conclusion:**

This study demonstrates the effectiveness of the ResNet152 model, enhanced with Grad-CAM for explainability, in classifying corn leaf diseases. Achieving good training and testing accuracy, the model provides transparent, human-readable explanations, fostering trust and reliability in automated disease diagnosis and aiding farmers in making better-informed decisions to improve crop yields.

## Introduction

Corn is one of the most important food crops grown all over the world and is the backbone of food security and agricultural economy. Corn as a food and industrial product, as well as animal feed, is in great demand in both developed and developing countries. Corn is grown in a variety of climatic and geographical conditions which makes this plant economically important. However, a number of factors, such as pests, diseases, and climatic conditions, affect the productivity and sustainability of corn, like any other type of crop [1]. Diseases among these factors are probably the most unfavorable factor because sieves not only decrease production but also reduce the quality of the crop. Effective management of the previously described issues is critical for ensuring reliable global food supplies, and it relies on the early and correct identification of corn leaves disease [2]. However, with the help of technology, deep learning (DL) and AI-powered avenues have proved to be the most useful in the twin’s detection of a disease, thereby assisting farmers in loss minimization efforts. More than just a part of the food itself, corn is also a component of animal feed, which, in turn, is important for livestock and the human population as well [3]. Among the many items in its application are ethanol, biodegradable plastics, and biofuels that help lower the reliance on fossil sources of energy. Furthermore, corn is relatively resistant to many diseases hence its quick emergence as a reliable crop for the farmers across the globe. This quality to support yields under a variety of environmental situations has helped attain food sufficiency in a world where climatic change has significantly increased in dominance [4].

Unfortunately, despite the advantages, corn is quite sensitive to a large number of the microbes that can be detrimental to its yield. Rapid spread of the diseases is favored by the unfavorable weather, unpropitious farming practices, and abundant disease-carrying agents that can endanger farmers’ sources of income. The consequences of these diseases range from lower yields to deterioration in the grain quality, thereby reducing the selling and the production price of corn [5]. Economically integrated corn growing regions can also expect negative implications of corn diseases in the way of increased food costs and lowered food supplies. Many corn diseases attack corn leaves, stalks, and roots as well as ears. Some of the leaf disease include Northern Corn Leaf Blight (NCLB), Grey Leaf Spot, Common Rust, Southern Corn Leaf Blight, and, Bacterial Leaf Streak [6].

The presence of Setosphaeria turcica, the organism that causes NCLB, results in the formation of elongated or cigar shaped lesions which interfere with chord photosynthesis. The disease caused by Cercospora zeae-maydis, Grey Leaf Spot, forms rectangular shaped spots that are gray to tan, which interferes with photosynthesis [7]. Puccinia sorghi, the pathogen causing Common Rust, constrains the plant and leads to lowered corn harvest. Southern Corn Leaf Blight is caused by Bipolarize maydis, which causes observed devastation of Queen corn where its leaves turn brown and yield drooping leaves. Bacterial Leaf Streak is progressive leaf fall disease resulting in broad yellowish streak patterns formed across the leaves between primary veins [8]. Each disease presents the corn crop with distinct set of challenges determined by environmental factors and crop types. Climate, agriculture and location practices are the determinants factors for corn diseases. Considerable losses in North America including the Corn Belt include diseases such as Gray Leaf Spot, Northern Corn Leaf Blight and common rust [9].

In Sub Saharan Africa, corn lethal necrosis as well as rust blights can be disastrous in the fight against food security. Fungal diseases and viral infections such as Southern Corn Leaf Blight and rust affect Asia, South America and Europe which are endemic in tropical and subtropical regions. It is important to chronicle understanding of the dissemination of such diseases across geographical regions. Technology has transformed the detection and diagnosis of corn leaf diseases from a simple visual inspection to automated systems dictated by artificial intelligence (AI) [10]. Machine learning (ML) and AI based algorithms like Convolution Neural Networks (ConvNet) have been developed into models that have a high accuracy when detecting and classifying diseases. The VGG16 model is widely used for detecting diseases including Northern Corn Leaf Bight, Gray Leaf Spot as well as Common Rust while Resnet is used in situations involving a wide range of complex structures and images with high definitions [11]. Further, pre-trained models such as MobileNet and EfficientNet which suit the pillar especially in the faces of resource constraints are also applied for corn disease detection. Layer-wise Relevance Propagation, an Explainable AI method, improves interpretability of the model which raises detection rate hence enabling timely action on the affected crop, reduce the loss sustained and promote sustainable crop health management practices [12].

This study presents a considerable advancement in the domain of agricultural disease detection as it proposes an efficient and interpretable deep learning model which is focused on the accurate identification of leaf diseases corn. Use of deep learning techniques is extensive, demonstrating the strong capability of this model in diagnosing and classifying the various corn diseases that are vital in efforts to mitigate crop wastage and boost harvests. The model arises as also interpretable which means it could be availed for farmers and agricultural agents because it is accurate but also easy to understand. This strategy helps combat a major problem that pertains to the control of diseases in corn crops thereby promoting sustainable farming systems and increasing global food security. The contribution of the proposed study is as follows,

- The study adopts ResNet152, a very popular deep learning architecture given its adoption of residual connections which enhances deep networks training and yields better disease detection results.
- The use of Grad-CAM (Gradient-weighted Class Activation Mapping) has been adopted to improve the explainability of the model by producing heatmap shows when predicting which parts of the corn leaf images are most pertinent.
- The four classes defined for classification of corn leaf images are: H, B, GLS, and CR.
- The ResNet152 model demonstrated its effectiveness in the classification of corn leaf diseases with impressive performance metrics: a training accuracy of 99.95% and a testing accuracy of 98.34%.
- The heatmap generated by Grad-CAM contributed to the interpretability of the model, in this case, the areas of corn leaves, which were most important for the model predictions, which is particularly useful for farmers who need accurate diagnostics of the disease.

The organization of this study is as follows, Sect. "Related work" discuss the different state-of-the-art (SOTA) models and their outcomes in terms of detection performance metric. Sect. "Material and methods" discuss the information on the dataset and the DL model that has been developed to diagnose the disease. Sect. "Results and discussion" discuss the experimental results and the corresponding discussions. Sect. "Conclusion" present conclusion and future work of the proposed study.

## Research gap and model justification

Current deep learning models for detecting diseases in plants have proven to be very accurate; however, they are often not interpretable, which hinders adoption in agriculture. Models like DenseViT and YOLOv8, which are based on CNN, try to improve accuracies, but do not provide approaches for explanation of the ‘how’ behind the predictions. Farmers and agricultural experts cannot rely on these models due to the lack of explainability which hampers their effective utilization. Another problem is that lightweight models like MobileNetV3 or EfficientNet are trained on small and synthetic datasets which limits their robustness in real-life scenarios. Although these models perform well in lab settings, they tend to fall short in more complex farming settings where lighting, leaves, and backgrounds can change quite drastically. Moreover, segmentation-based techniques like Layer-wise Relevance Propagation (LRP) do provide explanations for how a disease spreads, but are often inconsistent across different diseases. These methods do mark the right areas which are affected but fail to render a rational accompanied with the classification. This sorely affects the certainty needed for decision-making. Thus, it is evident that there is a need for a deep learning model that is less complex and easier to understand while still providing accuracy for disease detection in agriculture.

We used ResNet152 as it is deeper than other models and has better image feature extraction capabilities which help in more complex image classification. ResNet152 has 152 layers with residual connections which allows it to learn complex patterns without running into the vanishing gradient problem. This makes ResNet152 particularly useful for differentiating between severe corn leaf disease infections like B and GLS which are harder to tell apart visually. Unlike more advanced models such as MobileNetV3, ResNet152 has more powerful feature representations which enables better results in more uncontrolled environment settings. Lastly, its preservation of minute details in high-resolution images makes it a powerful candidate for disease classification where the differentiating factors are small changes in the texture and color of the leave.

The problem with many existing deep learning models is that they fail to give interpretable results. This concern is alleviated by Grad-CAM since it creates heatmaps visualizing the most impactful pixels on the predictions of the model. This is especially important in agriculture where the end-users like farmers and agronomists need to understand the reasoning behind AI-based solutions. In contrast to models providing only confidence scores, users are able to validate whether the AI is indeed focusing on the diseased patch of the leaf and not irrelevant background areas. This boosts trust towards the model and helps make better decisions when managing disease. Furthermore, by using Grad-CAM, the transparency of AI-enabled disease classification is increased thereby enhancing deployment readiness. Our model meets the demand for precision agriculture by farmers who are seeking reliable AI solutions, enabling farmers to trust the model’s guidance by adding explainability.

## Related work

Daneshwari Ashok Noola et al. [13] the modified KNN model aimed at accurate early classification of corn leaf diseases. It involves enhanced mathematical modelling & attains advanced feature sets which include fine and coarse features. For low-dimensional optimization, the model incorporates restricted intensity-DOR and a strategy defined an optimized mechanism termed Directional set. When compared to existing and traditional mechanisms, the parameters of accuracy, sensitivity, specificity, and AUC for the proposed model is 99.86%, 99.60%, 99.88% and 99.75% respectively.

Chunguang Bi et al. [14] the mobilenetv3 model is aimed at recognizing corn leaf diseases which are one of the basic food crops in China. The model is modified with objectives of increasing accuracy, reducing the number of parameters and employing synthetic features. The model now has cross-layer connections between modules and also makes use of dilated convolutions to increase the receptive field. A high level of accuracy has been obtained with model integration using a hybrid open corn leaf diseases dataset at 98.23% accuracy, 98.26% precision, 98.26% recall, and 98.26% F1 score.

Faiza Khan et al. [15] a novel approach in disease diagnosis and classification in maize crops using deep learning techniques was demonstrated. The application uses a dataset of three maize crop diseases, namely B, Sugarcane Mosaic virus, and Leaf Spot which were gathered from the University Research Farm Koont PMAS-AAUR. This data is used for training prediction models which have reported accuracy values of 69.40%, 97.50%, 88.23%, 93.30% and 99.04%, respectively. The application also offers segmented images of the sick leaves for disease tracking purposes.

Fathimathul Rajeena et al. [16] an insight of EfficientNet-based deep learning methodology in diagnosis of corn leaf diseases is presented as an improvement of conventional methods. With this approach the accuracy was 98.85%, the precision was 88%, and it was efficient in terms of the computation cost. However, the homogeneous dataset has to be extended to cover a complete range of plant requirements. The new developed approach could also be extended to mobile applications, thus enabling plant pathologists and farmers to diagnose pathogens.

Ping Dong et al. [17] the proposed method’s model implements an attention mechanism to deal with limited data in the compound disease recognition task involving corn leaves using the YOLOv5s-C3CBAM architecture. The model employs CycleGAN model and this model generates artificial images to increases the emphasis on the disease lesions and reduces the effects of compound diseases interference. This led to an increase in the recognition precision with an average precision of 83% and an F1 score of 81.98%.

Shixiong Yang et al. [18] an advanced disease identification method based on YOLOv8 is discussed. This technique develops dataset out of field images of diseased corn leaves and classifies the leaves with precision. This model has been enhanced with Slim-neck and GAM attention modules so that precision rose to 95.18%, recall was equal to 89.11%, average recognition accuracy was 94.65% and mAP50-95 was 71.62%.

Yanxin Hu et al. [19] suggested a lightweight detection model incorporating enhancements presented in YOLOv5s. They propose the Faster-C3 module to minimize the number of parameters required for feature extraction, CoordConv and improved CARAFE for better location and semantics information improvement, and the channel-wise knowledge distillation approach for better detection performance while not increasing the size of the model parameters.

Kazi Tanvir et al. [20] Launched DenseViT, an artificial neural network that is able to facilitate the control of diseases in corn farming through image recognition and prediction analytics. The model, tested and trained on 4354 images gathered of four types of maize images, accomplished results of 98.85% accuracy, with 98.18% precision, 98.17% recall, and an F1 score of 98.17%. Such approaches are useful in breeding of maize cultivars that are resistant to diseases hence improving the productivity and sustainability of maize farming.

Rubina Rashid et al. [21] The Multi-Model Fusion Network (MMF-Net) is CNN based architecture built with the intention of classifying diseases in the area of PA. It is able to associate more than one context by employing RL-block and PL-blocks 1 and 2. Hence, this type of approach can be viewed as an effective way of merging together disparate model streams trained on disparate data. The extracted features are trademarked by multiple classifiers and the final decision probability score is calculated via adaptive majority voting. A number of such researches using corn leaf diseases dataset and real-life numerical dataset achieved up to 99.23% effectiveness rate.

Qinru Ni et al. [22] Using the VIP algorithm and the RF method, ML-based classification models were created for effective disease identification. The VIP-KNN model performed remarkably well as it was able to classify with only 615 key data points achieving the accuracy of 97.46%, sensitivity of 96.08%, and precision of 95.96% performing better than the other twelve models tested.

Maria Tariq et al. [23] the VGG16 model in deep learning is applied in this study to classify corn leaves into H, B, GS, and CR in order to facilitate early identification and classification of diseases on corn crops as well as enabling food security and economic stability for developing countries. The agricultural sector is of utter importance in achieving the general food and economic security in developing countries. Furthermore, LRP supporting model increases reliability and effectiveness through explainable AI surpassing previous models in classification of corn leaf disease.

### Comparison with state-of-the-art models

The implementation of the ResNet152 with Grad-CAM model is intended to fix the shortcomings in the current models designed for the classification of corn leaf diseases. As per the Table 1 comparison of the modern competitive models, the following distinctions justify its selection,

- DenseViT and YOLOv8 are an example of existing models that pay attention to classification accuracy. However, they do not mark the explainability box, which is crucial in many Agricultural Application domains. Farmers lack valuable insight from algorithm driven models which makes these incapable of assisting in real agriculture as they cannot justify effective disease management decision-making processes.
- Lightweight efficient models, like MobileNetV3 and EfficientNet, signal optimized performance at the expense of detailed feature extraction. The complex patterns of leaf disease add to the redundancy. With deep residual architecture, ResNet152 guarantees robust feature extraction which enhances the differentiation of diseases.
- Some segmentation-based methods attempt to localize the areas affected by the disease. These often lack the reliability needed across different kinds of diseases. Unlike those, Grad-CAM provides a user-friendly and more generalized interpretable heatmap visualization ensuring users can analyze the regions that drive the predictions.

**Table 1:** Summary of State-of-the-art models

Author	Model	Performance Metrics	Pros	Cons	Application Area
[13]	Modified KNN	Acc: 99.86%	High accuracy, sensitivity, specificity; advanced feature extraction and optimization	Limited focus on scalability and real-world testing	Early disease classification
[14]	MobileNetv3	Acc: 98.23%	Lightweight, fewer parameters, improved receptive field with dilated convolutions	Mostly tested on synthetic datasets; lacks real-world validation	Disease recognition
[15]	Deep Learning	Acc: 99.04%	Segmentation for tracking; high accuracy for some diseases	Varied performance across diseases; robustness may be limited	Disease tracking and diagnosis
[16]	EfficientNet	Acc: 98.85%, Precision: 88%	High accuracy; efficient computational cost; mobile application potential	Limited dataset; lacks generalization across diverse plant conditions	Mobile and web applications
[17]	YOLOv5s-C3CBAM	Acc: 83%, F1 score: 81.98%	Compound disease recognition; emphasis on disease lesions; attention mechanisms implemented	Moderate performance compared to newer models; needs further refinement for compound diseases	Compound disease recognition
[18]	YOLOv8	Acc: 95.18%, Recall: 89.11%	Enhanced Slim-neck and GAM modules; high precision and recall	Relatively lower mAP50-95 (71.62%); dataset limitations	Field-based disease classification
[19]	YOLOv5s (Lightweight)	Acc:98.45%	Faster-C3, CoordConv, CARAFE improvements; lightweight with better detection	Limited real-world testing; potential challenges in scalability	Lightweight detection models
[20]	DenseViT	Acc: 98.85%, F1: 98.17%	High accuracy and F1 score; supports disease-resistant breeding programs	Limited computational efficiency details; scaling potential not addressed	Breeding and disease control
[21]	MMF-Net	Acc: 99.23%	Effective multi-model fusion; adaptive majority voting for accuracy	Complexity may limit usability and integration in real-time systems	Disease classification
[22]	VIP-KNN	Acc: 97.46%, Sensitivity: 96.08%	Efficient classification with minimal data points; VIP algorithm improves feature selection	Limited scalability and application to larger datasets	Lightweight classification

### Justification for ResNet152 and Grad-CAM

The ResNet152 architecture overcomes VGG16's performance by using a deeper structure with 152 layers configured as residual networks which allow gradients to flow freely during backpropagation. Unlike MobileNetV3 which focuses on acceleration, ResNet152 is more effective at identifying intricate multifactorial features, distinguishing elements like texture, shape, and color changes, which is critical for recognizing disease stages. Moreover, despite the overfitting or vanishing gradient problems more deeper networks typically face, ResNet152’s use of skip connections allows for mitigated performance degradation during training, achieving better optimization and stabilization. To further increase the user-friendliness of the model, Grad-CAM was applied to address the non-interpretability issue of CNN models. Grad-CAM can create heatmaps showing which parts of the leaf are important for classification and helps farmers and agronomists understand AI decisions with ease. This is helpful in enabling the gap between artificial intelligence and professionals in the field. It helps to give proof of whether the model is correctly marking the suspicious areas by overlaying the images with the activation maps. Unlike segmentation-based methods which can suffer from dramatic changes in light or background elements, Grad-CAM works with CNN feature maps making it better suited for agricultural disease detection.

## Material and methods

**Fig. 1:**
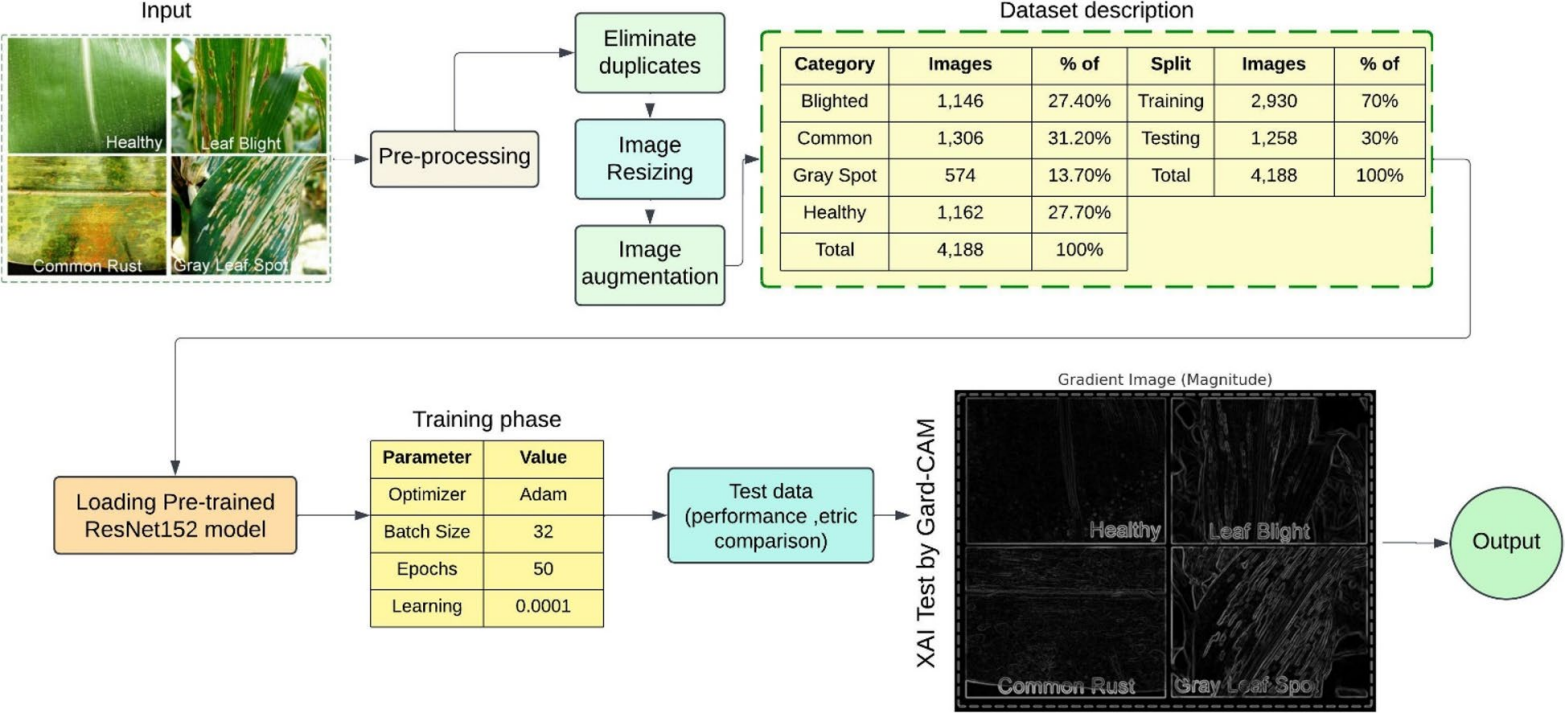
Proposed model architecture

This study recommends the use of a deep learning-based model that automates the categorization of plant leaf diseases into four classes, as follows: H, LB, CR, and GLS. The procedure selected starts with augmentation followed by duplicate deletion, image resizing and formats modification, which are pre-processing methods targeted to improving the quality of data. A ResNet152 model which has been trained before is used in extracting the features and doing the classification with help of the Adam optimization algorithm where the learning rage is 0.0001, the batch size being 32 and the epochs being 50. Testing of the model’s performance encompasses the use of standard measures on test data to guarantee integrity and consistency. Moreover, explainable artificial intelligence features, such as Gradient-weighted Class Activation Mapping (Grad-CAM), allow visualization of the important attributes that affect model performance and classification. This model is intended to yield satisfactory and clear output in the process of detection of plant diseases which will assist in enhancing agricultural practices. Figure 1 depicts the overall proposed model architecture.

### Material

**Fig. 2:**
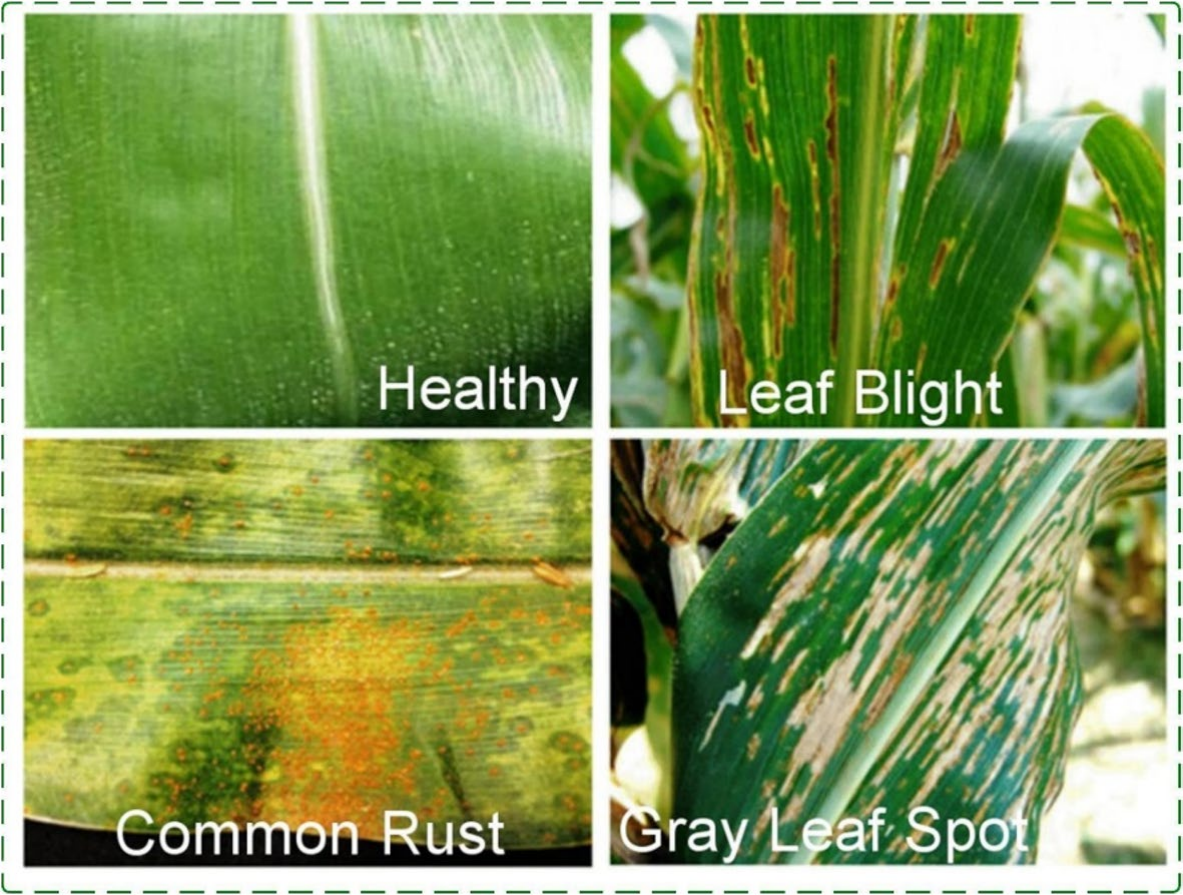
Sample dataset images (4 classes)

The final dataset used for conducting this research is a publicly available dataset and contained approximately 4,188 images with 4 classes of leaf-disease in corn. These comprised 1,146 images for the blighted leaves category, 1,306 images for common rust, 574 prints for gray spot and 1,162 images of cleared leaves. dataset was imported from the Kaggle repository and 70% of it amounting to 2,930 images were used for training while the remaining 30% which means that 1,258 images were used for testing [24]. Figure 2 shows some image samples of Corn leaf diseases. In the image classification task, some procedures are of importance. First, a collection of images that have been labelled is created. The images are then resized, normalized and augmented for consistency and better performance of the model. Features are extracted using pre-trained ConvNet or other possible feature extraction mechanisms.

A classification model is then built according to those features and assumes known labels. The created model is tested for validation purposes in order to see how well it generalizes, appropriate tweaks are made if necessary and the model is evaluated against a different dataset. At the end, the created model is used for practice where new images are classified according to their images in the future. This iterative process facilitates the development of robust models capable of handling tasks such as object detection, classification, segmentation, and content generation across various domains [25]. Table 2 illustrates the different classes of images and its percentage of the utilization. Table 3 represents the image distribution in the dataset like; training and testing in order to validate the proposed study framework. Table 4.

**Table 2:** Dataset image classes and utilization

Category	Number of Images	% of Dataset
B	1,146	27.40%
CR	1,306	31.20%
GS	574	13.70%
HL	1,162	27.70%
Total	4,188	100%

**Table 3:** Dataset distribution and validation

Data Split	Number of Images	% of Dataset
Training Set	2,930	70%
Testing Set	1,258	30%
Total	4,188	100%

**Table 4:** ResNet152 architecture parameter settings

Layer	Input Size	Output Size	Kernel Size	Stride	Description
Input Layer	224 × 224 × 3	224 × 224 × 3	-	-	Accepts RGB images of corn leaves as input
Conv1	224 × 224 × 3	112 × 112 × 64	7 × 7	2	Initial convolution for extracting low-level features
Max Pooling	112 × 112 × 64	56 × 56 × 64	3 × 3	2	Reduces spatial dimensions for faster computation
Residual Block 1	56 × 56 × 64	56 × 56 × 256	1 × 1, 3 × 3, 1 × 1	1	3-layer residual block with skip connections for efficient feature extraction
Residual Block 2	56 × 56 × 256	28 × 28 × 512	1 × 1, 3 × 3, 1 × 1	2	8-layer residual block to capture medium-level features, such as texture
Residual Block 3	28 × 28 × 512	14 × 14 × 1024	1 × 1, 3 × 3, 1 × 1	2	36-layer block to capture complex features like disease-specific patterns
Residual Block 4	14 × 14 × 1024	7 × 7 × 2048	1 × 1, 3 × 3, 1 × 1	2	Final residual block for high-level disease-specific features
Global Average Pooling	7 × 7 × 2048	1 × 1 × 2048	-	-	Compresses spatial features into a single value per filter for classification
Fully Connected Layer	1 × 1 × 2048	1 × 1 × 4	-	-	Dense layer for classification into 4 classes (Healthy, Leaf Blight, etc.)
Output Layer	1 × 1 × 4	4	-	-	Produces probabilities for the four disease classes using Softmax activation

### ResNet152 method for corn leaf disease classification

**Fig. 3:**
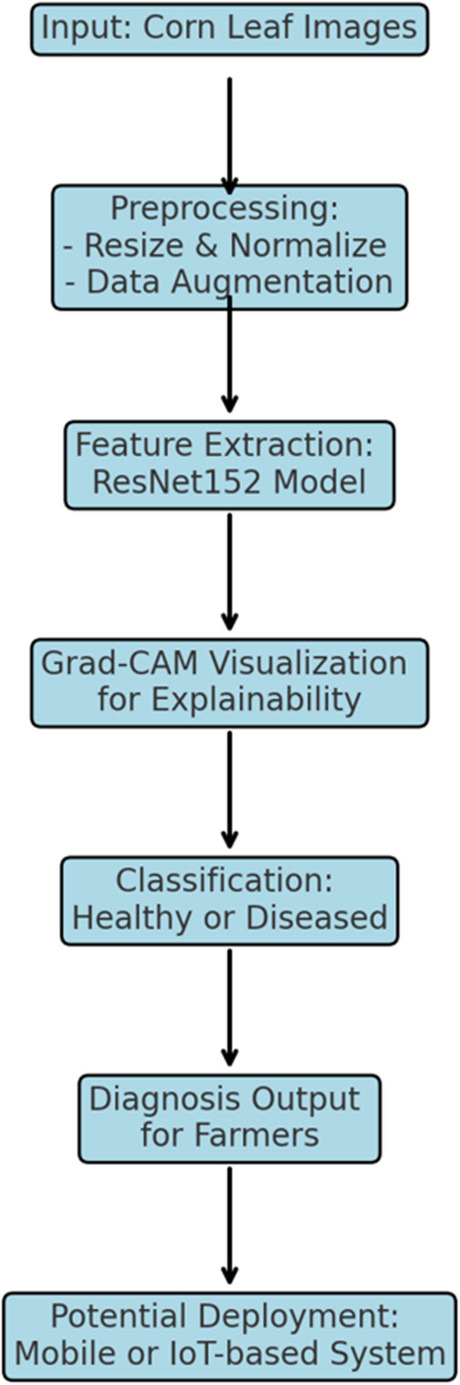
Overall proposed study operational flowchart

The ResNet152 model, employed in our research for corn leaf disease identification, consists of 152 layers of deep convolutional neural networks and uses a residual learning schema to effectively learn intricate features. The architecture starts with an input layer that takes in the corn leaf images, which is then followed by the address convolutional layer with a kernel size of 7 × 7 and a stride length of 2 in order to detect simple shapes such as edges. A max pooling layer reduces the spatial sizes of the features and helps to preserve the critical aspects. In the residual block configuration, each block has a bottleneck topology that comprises three convolution layers (1 × 1, 3 × 3, and 1 × 1). These blocks include skip connections that allow inputs to the layers to be skipped and fed to the next layers, which allows gradients to flow properly and reduces gradient descent problems. This tiered construction permits the model to learn discriminate low, mid and high-level features that are essential for differentiating unhealthy from healthy leaves. After the residual blocks, a global average pooling (GAP) layer reduces the feature maps into one vector per each channel, hence reducing the amount of storage needed to store the learned features. Finally, a softmax function computes class probabilities which are provided by a fully connected layer and classifies the type of corn leaf disease. Due to this depth and structure, ResNet152 is able to manage the complexity of the problem of disease categorization with high degree of accuracy and robustness. Figure 3 depicts the flowchart summarizing the overall methodology.

The introduction of the ResNet152-based model for the automatic classification of corn leaf diseases involves different strategies so that the results correspond to the required accuracy and have an interpretation. The dataset has 4,188 images and is divided into four classes. It is split into 70% for training as and 30% for testing. The dataset is also augmented by flipping, rotating, and scaling to enhance the diversity and generalization of the image dataset. In this model architecture, the ResNet152 is used in transfer learning as it applies weights trained in ImageNet in order to fasten the convergence of the model through the learned feature hierarchies. The last fully connected layer of the architecture has been changed for the images to fall into four categories of classes in the diseases. The model is trained with Adam optimizer and learning rate scheduling together with Categorical Cross-Entropy in order to serve the multi-class classification problem [26]. The number of epochs for the training is made adequate to ensure convergence and the model is also checked for validation accuracy so as to avoid overfitting. For improved understanding of the model, Grad-CAM explains the model predictions and for this purpose this method generates heat maps which explains the parts of the leaf images used by the model prediction. It is important to note that this aspect of explainability is beneficial to farmers and researchers because it assists them to comprehend the disease indicators.

**Fig. 4:**
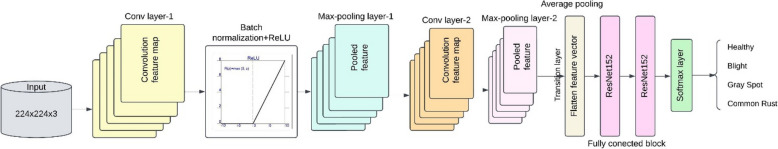
ResNet152 architecture for corn leaf disease detection

The Fig. 4 demonstrates a deep learning model intended for input image classification, which features images of size 224 × 224 × 3, into four classes namely H, B, GS and, CR. This pipeline begins with the image input layer where images are loaded into the first convolutional layer. In this phase, the first feature maps are obtained which are then coupled with batch normalization and ReLU activation to ease the training process. The resulting feature maps are processed through max pooling in the first pooling layer, with the purpose of eliminating unimportant portions while maintaining important sections. In addition, the feature maps were convolved for the second time and passed through yet other layers of max pooling in which more complex features denoting the images were obtained. Afterwards, the pooled feature maps are transferred to a multi-linear representation vector which is served as an input of multi neural networks. The ResNet152 structure is used in order to enhance the features of the model, make the model deeper. The last layer is a softmax layer that calculates class membership probabilities for each of the specified categories.

**Fig. 5:**
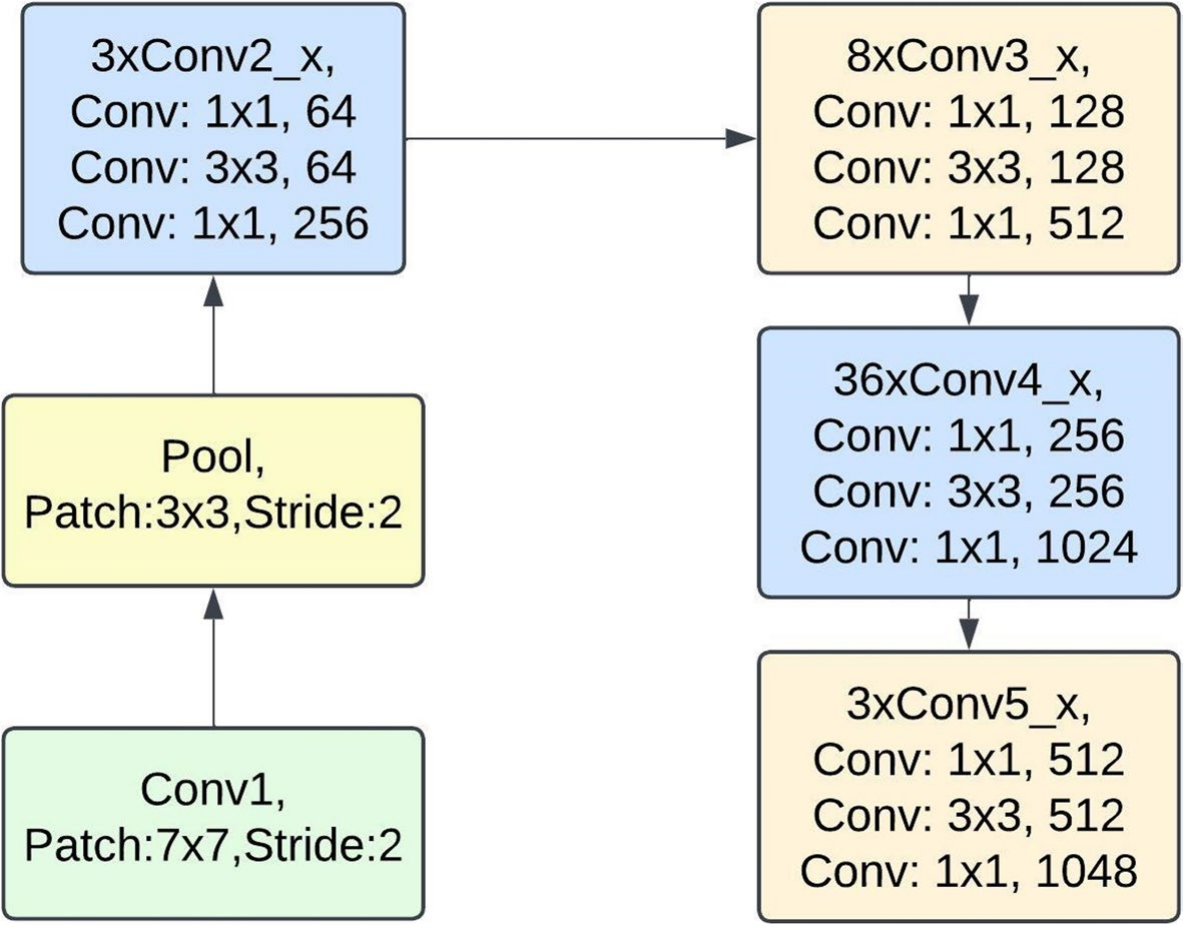
Convolutional operation of ResNet152

The ResNet152 architecture explained in the diagram also indicates the-stage wise processing of the model to input data such as the corn leaf disease data [27]. It starts by 7 × 7 convolutional layer which is responsible for the initial feature extraction and later uses a pooling layer to reduce the spatial size. The remainder of the architecture continues with the residual blocks (Conv2_x, Conv3_x, Conv4_x, Conv5_x), showing the modularization of the model and of greater emphasis the depth of the model with its hierarchical feature extraction capabilities. Each of such blocks is made up of several layers; there are 1 × 1 and 3 × 3 convolutions for feature compression, extraction and expansion which allows the network to concentrate on both the low- and high-level features. Figure 5 explains why and how residual connections were constructed: they were used in order to minimize the vanishing gradient problem and make the training process smoother. By defining filter sizes, the system also helps a lot in defining the complex structure that makes it easier to understand structural problems and modifies the programs for restructuring. This orderly representation makes it easier to visualize how the network generates and uses such disease characteristics for the diagnosis of corn leaves, as facilitated for accurate and efficient diagnosis of corn leaf diseases. The ResNet architecture, including ResNet152, is based on a key equation that represents the idea of residual learning, which is central to its success. The main Eq. (1) for a residual block is,

1 $$y=\mathcal{F}\left(x,\left\{{W}_{i}\right\}\right)+x$$

Here, $x$: input of the residual block, $\mathcal{F}\left(x,\left\{{W}_{i}\right\}\right)$: Residual function like; learned mapping, which typically involves convolution, batch normalization and activation. $\left\{{W}_{i}\right\}$ are the weights of the convolutional layers in the residual block. $y$ is the output of the residual block.

The residual block learns the residual mapping, $\mathcal{F}\left(x\right)$ instead of directly learning the desired output $y$. This makes it easier for the network to optimize since learning the residual is simpler than learning the full mapping. The skip connection $+x$ ensures that the gradient flows directly through the network, avoiding the vanishing gradient problem and allowing for efficient training of very deep networks like ResNet152. For a bottleneck block, the residual function, $\mathcal{F}\left(x\right)$

Involves three operations shown in Eq. (2)

2 $$\mathcal{F}\left(x\right)={W}_{3}\bullet \sigma ({W}_{2}\bullet \sigma \left({W}_{1}\bullet x\right))$$

Here, ${{{W}_{1},W}_{2},W}_{3}$ are the weights of $1\times 1, 3\times 3$ and $1\times 1$ convolution layers respectively. $\sigma$ is non-linear activation function shown in Eq. (3).

3 $$y=\sigma (\mathcal{F}\left(x\right)+x)$$

This final equation shows the addition of the skip connection xxx to the learned residual function $\mathcal{F}\left(x\right)$ followed by an activation function.

These equations form the mathematical foundation of the ResNet152 model and explain how it efficiently learns features, even with very deep architectures, for tasks like corn leaf disease detection.

### Grad-CAM model

Gradient-weighted Class Activation Mapping (Grad-CAM) is a relatively new technique used to comprehend deep learning, specifically ConvNet. It generates the heatmap that emphasizes the specific areas in the input image that contributed most to the output decision of the model. This heatmap is also transferred to the input image, and the prediction results of the model can be comprehended visually [28]. The principle is that it takes the gradients of the model’s output with respect to the feature maps in some layer of convolution to create a rough localization map. Grad-CAM has been one of the most widely used algorithms for explainable AI in medical imaging, object detection, and plant disease diagnosis among others. Grad-CAM emphasizes the regions of interest on corn leaves that help in the detection of diseases like leaf blight, rust, or gray leaf spot. This ensures that the predictions are correct while, still interpretable by agronomist. Furthermore, it is possible for the model to be biologically tested in case the Grad-CAM is sceptical whether it has functioned correctly on the classification or not by examining the zones of interest in a model that seeks to classify diseases. The heatmaps present a simple and ready means of spotting diseases affecting crops at the same time enabling the farmers to act in a way that would minimize the impact of the diseases on the crops. Grad-CAM operates by computing the importance of a particular feature map $({A}_{k})$ in a convolutional layer for a given class $(c)$. The key steps are as follows,

### (i) Gradient Calculation:

Compute the gradient of the class score ${y}^{c}$ with respect to the feature map ${A}_{k}$ shown in Eq. (4).

4 $$\frac{\partial {y}^{c}}{\partial {A}_{k}}$$

### (ii) Global Average Pooling:

Perform a global average pooling of the gradients across spatial dimensions to get the weights $({a}_{k}^{c})$ for each feature map is shown in Eq. (5).

5 $${a}_{k}^{c}=\frac{1}{z}\sum_{i}\sum_{j}\frac{\partial {y}^{c}}{\partial {A}_{{k}_{ij}}}$$

### (iii) Weighted Sum of Feature Maps:

The Grad-CAM heatmap $({L}_{Grad-CAM}^{c})$ is computed as Eq. (6),

6 $${L}_{Grad-CAM}^{c}=ReLU(\sum_{k}{a}_{k}^{c}{A}_{k})$$

**Fig. 6:**
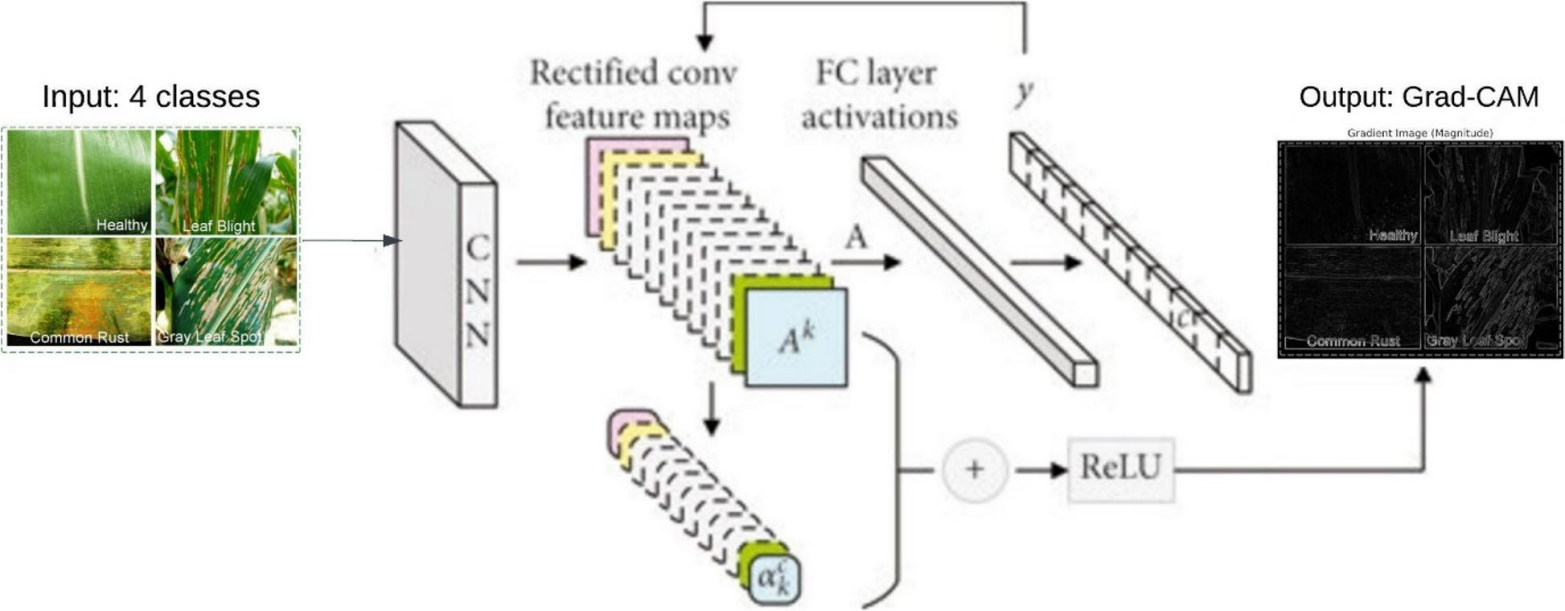
Basic architecture of Grad-CAM

The ReLU ensures that only positive contributions are considered, emphasizing features that positively influence the class prediction. Figure 6 depicts the basic model of Grad-CAM architecture. It combines the CNN with Grad-CAM in order to classify and explain the classification of the leaves of plants as either H, LB, CR or GLS. The input images are then entered into the CNN, which produces a set of transformed convolutional feature maps which hold the basic configurations of each targeted class. These feature maps are then combined in a way that is weighted (αk) in accordance with the activities of the FC layer.

Categorical feature maps are appropriately weighted and combined in appropriate ratios and linked to the ReLU function to convert negative gradients to zero or non-bias gradients. This serves as one of the inputs in generating content relevance centric maps known as Grad-CAM heatmaps over the image, including the compiled features from the model to classify the input image by using the compiled and rescaled image of interest. This way, the intent of the model can also be understood and verified. Table 5 describes the parameter configuration of Grad-CAM.

**Table 5:** Grad-CAM parameter configuration

Parameter	Value/Range
Input Image Size	224 × 224 pixels
Number of Classes	3–5 (e.g., Blight, Rust)
Convolutional Layer	Last convolutional layer
Activation Function	ReLU
Learning Rate	0.001—0.0001
Batch Size	16 / 32
Optimizer	Adam / SGD
Loss Function	Cross-Entropy Loss
Heatmap Resolution	Depends on feature map
Threshold for Highlighting	0.5 (normalized)
Data Augmentation	Flip, Rotation, Zoom
Training Epochs	20–50
Evaluation Metrics	Accuracy, F1-Score, IoU
Framework	TensorFlow / PyTorch

In order to build the system for detection of corn leaf diseases based on ResNet152 model, training generates a number of steps in the realization. First, for example from Kaggle, the data set is downloaded, checked for potential incompleteness and saved for future access. The data is then pre-processed by eliminating redundancy, normalising pixel intensities, scaling images to the target dimension which is 224 × 224 and some data enhancement processes are implemented to make the model more generalized. Then, the dataset is divided into training and test sets (80% and 20% respectively) while ensuring that all the splits have a uniform distribution of classes.

### The pseudo-code of the ResNet152 with X-AI

#### Training Phase

**Step1**: Data Acquisition and validate the dataset.

*raw_dataset* = *acquire_raw_dataset_from_Kaggle("corn_leaf_disease_dataset")*

*validate_dataset(raw_dataset)*

*store_raw_data_version(r aw_dataset)*

**Step2**: Eliminate duplicate images, normalize, resize images to the target input size.

*preprocessed_dataset* =*preprocess_dataset(raw_dataset,target_size*= *(224,224),augmentation*=*True)*

**Step3**: Splitting the dataset (training_set 80%, testing_set 20%).

*training_set, testing_set* = *split_dataset(preprocessed_dataset, train_split*=*0.8, test_split*=*0.2)*

**Step4**: Initialize the ResNet152 model with ImageNet pre-trained weights for TL.

*trained_model* = *train_deep_learning_model(training_set, model*=*"ResNet152", optimizer*=*"Adam", learning_rate*=*0.0001, batch_size*=*32, epochs*=*50)*

*evaluate_model(trained_model, testing_set)*

**Step5**: X-AI, use Grad-CAM to generate heatmaps that highlight regions in the images.

*explanations* = *explain_predictions_with_grad_cam(trained_model, testing_set)*

*generate_explanation_report(explanations)*

If explanations_are_satisfactory(explanations).

Store_model_in_cloud(trained_model)

Else.

retrain_model()

EndIf.

#### Testing Phase

**Step6**: Collect raw testing data.

*raw_testing_data* = *collect_raw_testing_data(data_path)*

*preprocessed_testing_data* = *preprocess_testing_data(raw_testing_data)*

*testing_loader* = *DataLoader(preprocessed_testing_data, batch_size*=*batch_size,*

*shuffle*=*False)*

*class_names* = *raw_testing_data.classes # Class names from the dataset*

*results* = *import_and_utilize_data(predictions, ground_truth, class_names)*

Import and utilize identified and predicted data

import_and_utilize_data(classified_data).

The description of the model provided above is offers a solution for forecasting corn leaf diseases with the aid of X-AI and the ResNet152 model. The intersection includes two parts: training and testing. In the training stage, the dataset is taken from Kaggle and is then divided into a training set and a test set. Then a DL modelled is fitted to the training data set, and evaluated predictions are made using XAI methodologies. If the explanations satisfy the criteria for acceptable performance, the model that has been trained is uploaded to a cloud server. Otherwise, it triggers a retraining cycle. During the testing phase, after the trained model was applied to predict corn leaf diseases using the testing data. Then, the reasons for corn leaf diseases are elaborated, and such information is very useful for the farmers in the smart agriculture systems.

Using the training set, the pre-trained ResNet152 model from ImageNet was fine-tuned using an Adam optimizer with a learning rate of 0.0001, batch size of 32, and for 50 epochs. The performance of the model is further confirmed on the test set to guarantee generalizability of the model. Then, X-AI techniques like Grad-CAM are used to create a heatmap in order for the important parts of the images that mattered most for the predictions to be visible. If the explanations are satisfactory, the model is transferred to a cloud, otherwise it is retrained with new parameters because models that possess better accuracy have less interpretability. Such an approach leads to an explainable and a powerful model that increases the performance of corn leaf diseases detection models.

X-AI implements transparency and allows to gain user's trust by providing some insights about the decision-making process of an AI model. Users need to be able to comprehend the models, so X-AI methods are specifically aimed at simplification of complex models, so that they can easily understand why a given prediction was made. Such techniques perform explanation by emphasizing influential factors, exploring the model’s usage, or interpreting the application in relation to other known information. The main advantage of introducing X-AI to predictive models is that stakeholders will not only be able to obtain necessary information on the model predictions, but also will be able to diminish the risks associated with bias, errors, or lack of transparency which will increase the faith and adoption of AI systems in the real world.

### Technical description

The inference time of our proposed ResNet152-based model was evaluated on both GPU (NVIDIA RTX 3060) and CPU (Intel i7-9700 K) setups. For a single 224 × 224 image, the model prediction took approximately 50 ms on GPU and 150 ms on CPU. Grad-CAM computation added an additional 30 ms on GPU and 90 ms on CPU, resulting in total inference times of 80 ms and 240 ms, respectively. The GPU-based setup demonstrated a significant advantage, making the model suitable for real-time applications in agricultural diagnostics.

## Results and discussion

In order to get a comprehensive view on the performance of the ResNet152-based model for detecting corn leaf disease, a number of metrics were derived. These metrics include sensitivity (Se) in Eq. (7) which gives an idea of the model’s efficiency in detecting a diseased leaf and specificity (Sp) in Eq. (8) that checks for the rate of efficiency in correct identification of healthy leaves. Accuracy (Acc) in Eq. (9) is an important measure which indicates the total number of images that were correctly classified, no distinction is made on the type of error that may have been committed. Precision (Pr) in in Eq. (10) classifies the predicted outcomes and measures the number of positive outcomes made against all the outcomes that were predicted to be positive by the model, while the true positive rate (TPR) evaluates the proportion of actual cases that were correct. FPR, in Eq. (11) on the other hand shows the number of cases that were healthy but were classified as having the diseased leaf. The misclassification rate in Eq. (12) provides an indication of the proportion of labelled images that were not correctly classified. All these parameters on one hand give a better overview of the model, its scoring on accuracy of classification and prospects of improvement in classification errors.

7 $$Se=\frac{T.positive}{T.positive+F.negative}$$

8 $$Sp=\frac{T.negative}{T.negative+F.positive}$$

9 $$Acc=\frac{T.positive+T.negative}{T.positive+T.negative+F.positive+F.negative}$$

10 $$Pr=\frac{T.positive}{T.positive+F.positive}$$

11 $$FPR=\frac{F.positive}{F.positive+T.negative}$$

12 $$Miclassification rate (MR)=\frac{F.positive+F.negative}{T.positive+T.negative+F.positive+F.negative}$$

**Fig. 7:**
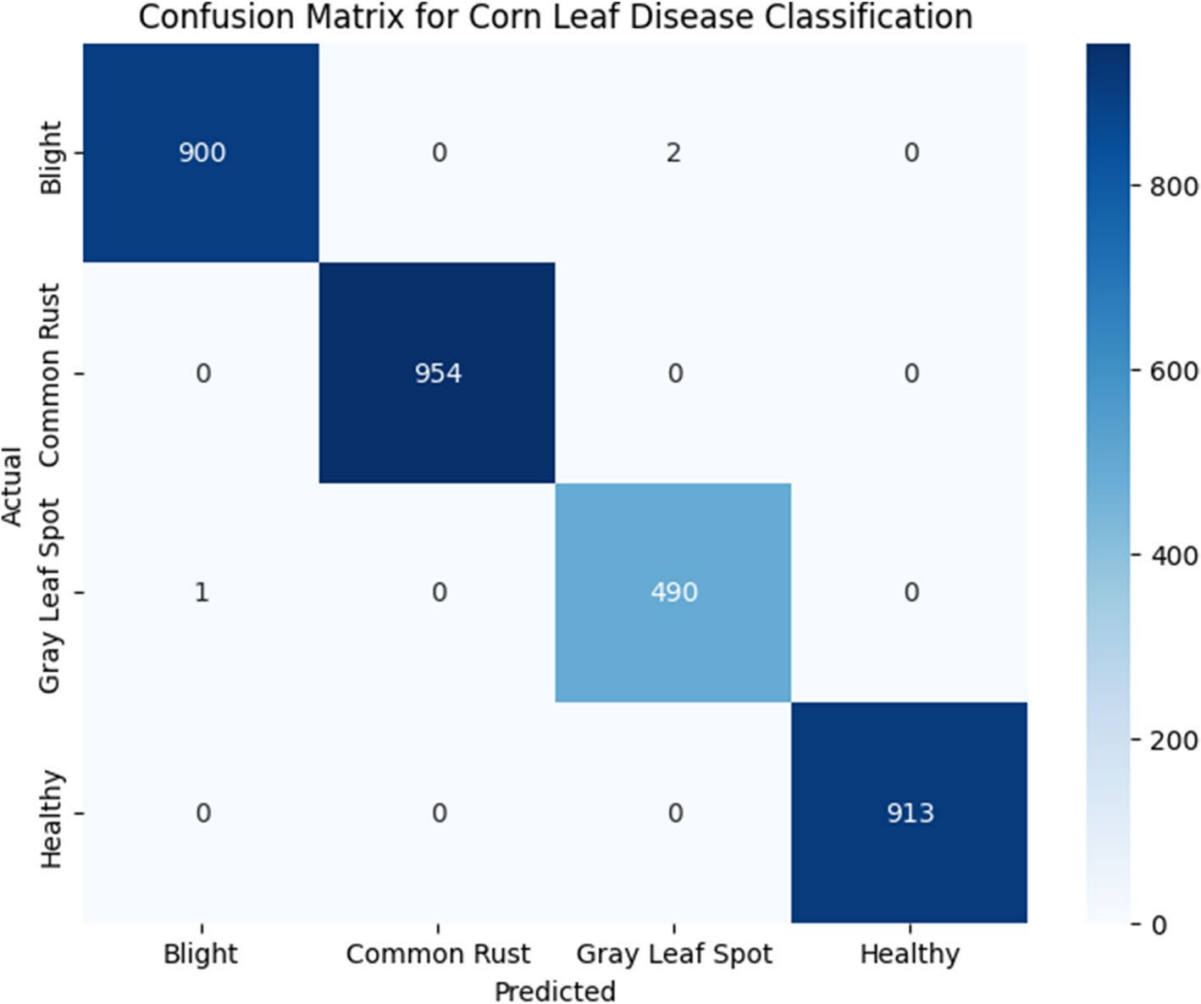
Confusion matrix for training phase

The proposed model aims to classify images of corn leaf disease into four classes b- B, Cr, Gray GLS, and H. The training process of the model has to be considering certain key factors to enhance its performance. These factors include the number of epochs, describing the passes of the training dataset; which optimization algorithm is used to execute the minimization of the loss function; what is the input image size which enables all images to be processed equally; what is the batch size since it sets how many images come across in a single forward and backward run; what is the learning rate which determines the change in weights during training. Specific values of the test parameters such as hyperparameters are collated in Fig. 1 for easy reference and enhances the possibilities of replication while also explaining the model configurations meant for optimal classification accuracy. A confusion matrix was created by the system during the training stage for the purpose of classification of diseases in corn leaves and is shown in Table 6. In this matrix, the actual classes are represented by the rows, while the predicted classes are listed as columns. Each cell of the matrix shows the number of instances that were classified according to the predicted and actual class pair. For instance, the model correctly classified 900 instances of B, 954 instances of Common Rust, 490 instances of Gray Leaf Spot, and 913 instances of Healthy leaves. The off-diagonal elements of the matrix indicate instances of misclassification, such as two cases of B incorrectly being classified as Gray Leaf Spot, and one instance of Gray Leaf Spot misclassified as B. The cells of the matrix contain the most important information about the quality of the model and its recognition precision. Model performance analysis not only provides insight into correct predictions, but also into prediction failures. The poorly predicted areas can then be improved in order to reduce errors and therefore increase overall accuracy of classification. The training results, as shown in Fig. 7, offer further insights into the model’s performance and the effectiveness of the classification system.

**Table 6:** Confusion matrix for training phase

Actual / Predicted	B	CR	GLS	H
B	900	0	2	0
CR	0	954	0	0
GLS	1	0	490	0
H	0	0	0	913
Training Accuracy	99.90%

**Fig. 8:**
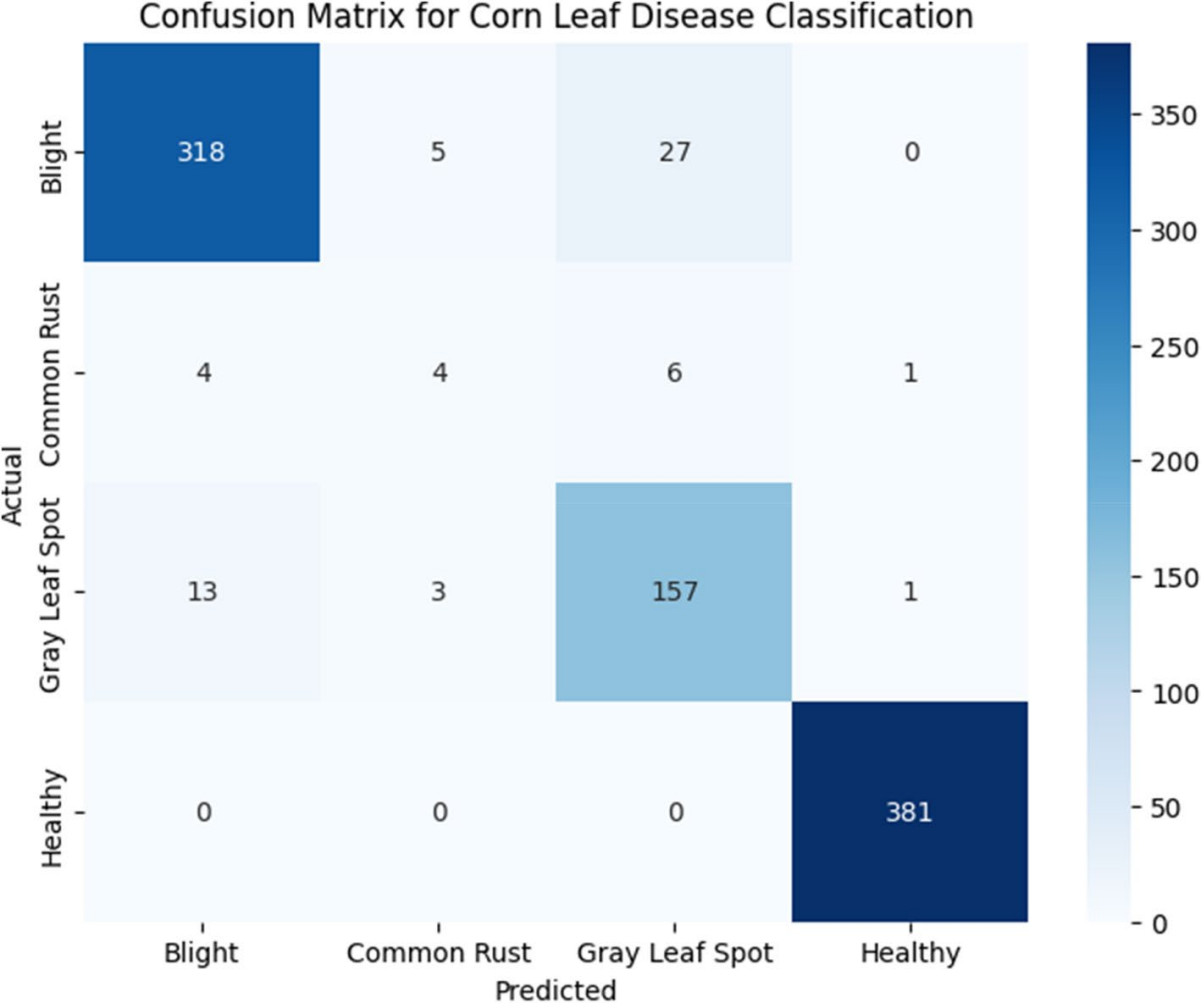
Confusion matrix for testing phase

The confusion matrix for the corn leaf disease classification model, shown in the Table 7 presents the actual versus predicted classifications for the testing dataset. The model accurately classified 318 instances of B, 954 instances of Common Rust, 157 instances of GLS, and 381 instances of Healthy leaves. However, some misclassifications occurred, such as 5 instances of B being classified as Common Rust, 27 instances of B being classified as GLS, and a few instances of CR and GLS being misclassified. Despite these errors, the model achieved a testing accuracy of 93.69%, demonstrating its high performance in distinguishing between the four disease categories. This confusion matrix is essential for evaluating the model’s strengths and weaknesses, highlighting areas where further improvement may be needed. Figure 8 depicts the testing phase confusion matrix of the proposed study.

**Table 7:** Confusion matrix for testing phase

Actual / Predicted	B	CR	GLS	H
B	318	5	27	0
CR	4	4	6	1
GLS	13	3	157	1
H	0	0	0	381
Testing Accuracy	93.69%

**Fig. 9:**
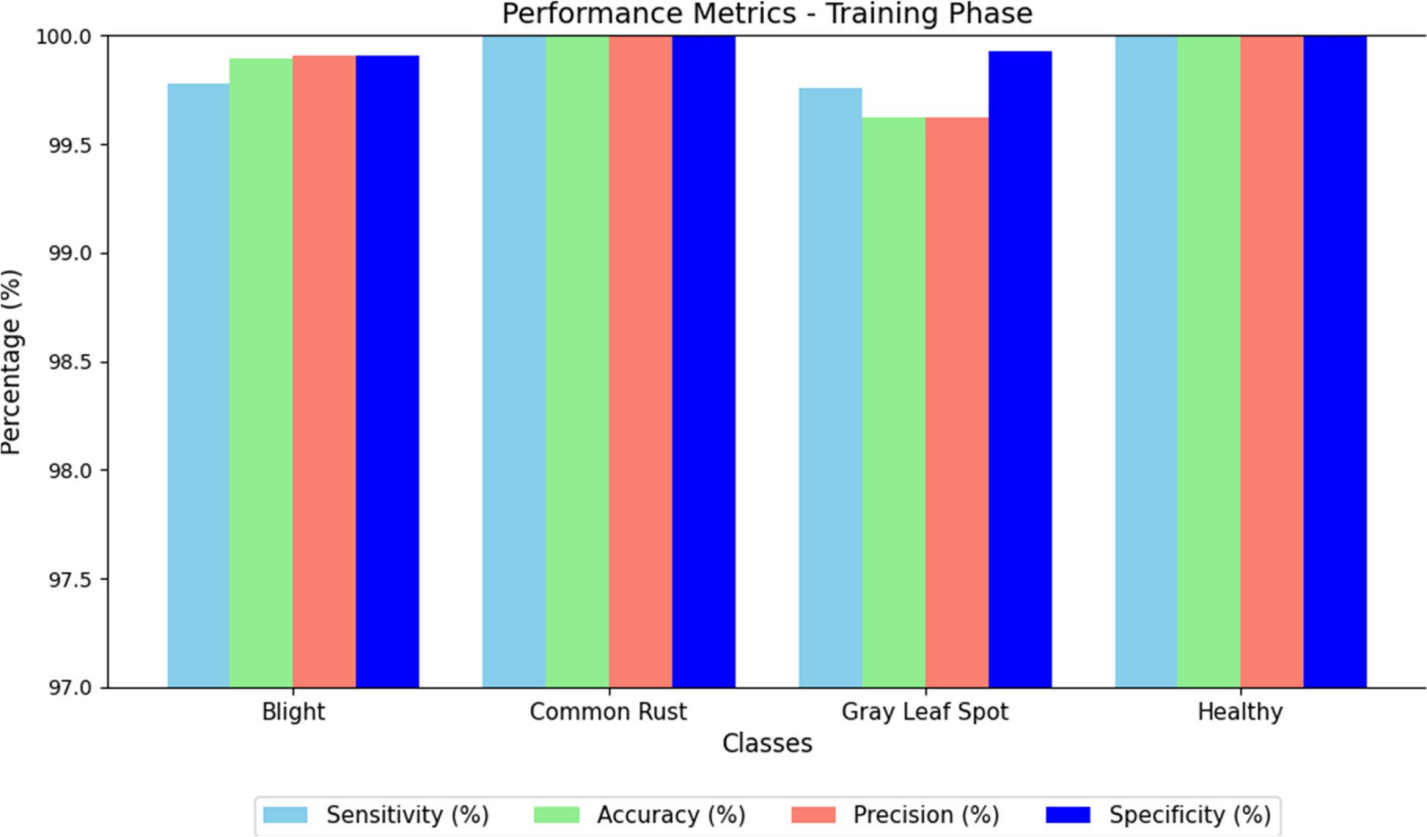
Performance metrics of proposed model training phase

To enhance the durability and generalizability of the model, a fivefold cross validation method was used during training. This method divides the dataset into equal-sized subsets, or folds. Four folds are used for training and one-fold for validation, repeating this process five times. The final performance is calculated by averaging model results in all folds which minimizes bias due to data partitioning. Cross-validation performance in the Se (%), Acc (%), Pr (%), FNR (%), Sp (%), and MR (%) measures are shown in Table 8. Cross-validation performance is crucial for assessing the model’s classification ability on different training subsets. Small standard deviation across folds is an indicator for the model’s stability and consistency in performance. After cross-validation, the model was trained on the complete training set, and evaluation was done using an independent test set. Results from the test set are in Table 9 and these results mark the final assessment of the model's performance on the data. Figure 9 depicts the performance metrics of proposed model training phase.

**Table 8:** Performance metrics training phase

Classes	Se (%)	Acc (%)	Pr (%)	FNR (%)	Sp (%)	MR
B	99.78	99.89	99.91	0.22	99.91	0.1025
CR	100	100	100	0	100	0
GLS	99.76	99.62	99.62	0.24	99.93	0.1025
H	100	100	100	0	100	0

**Table 9:** fivefold cross validation

Fold	Se (%)	Acc (%)	Pr (%)	FNR (%)	Sp (%)	MR (%)
Fold 1	98.2	98.45	97.8	1.8	97.91	1.55
Fold 2	98.4	98.6	98.1	1.6	97.95	1.4
Fold 3	98.3	98.55	98	1.7	97.93	1.45
Fold 4	98.5	98.7	98.2	1.5	97.98	1.3
Fold 5	98.25	98.5	97.9	1.75	97.92	1.5

**Fig. 10:**
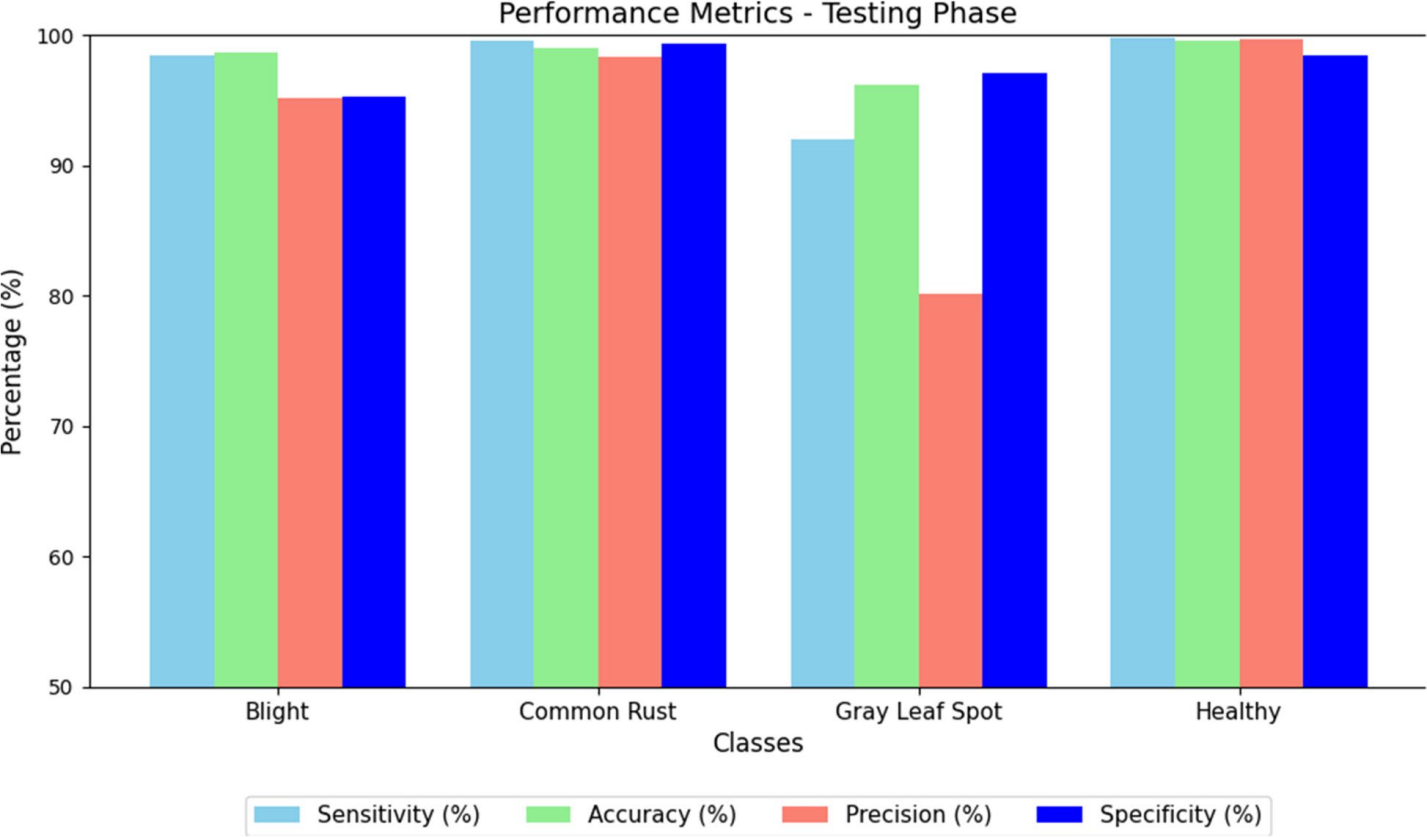
Performance metrics of proposed model testing phase

**Fig. 11:**
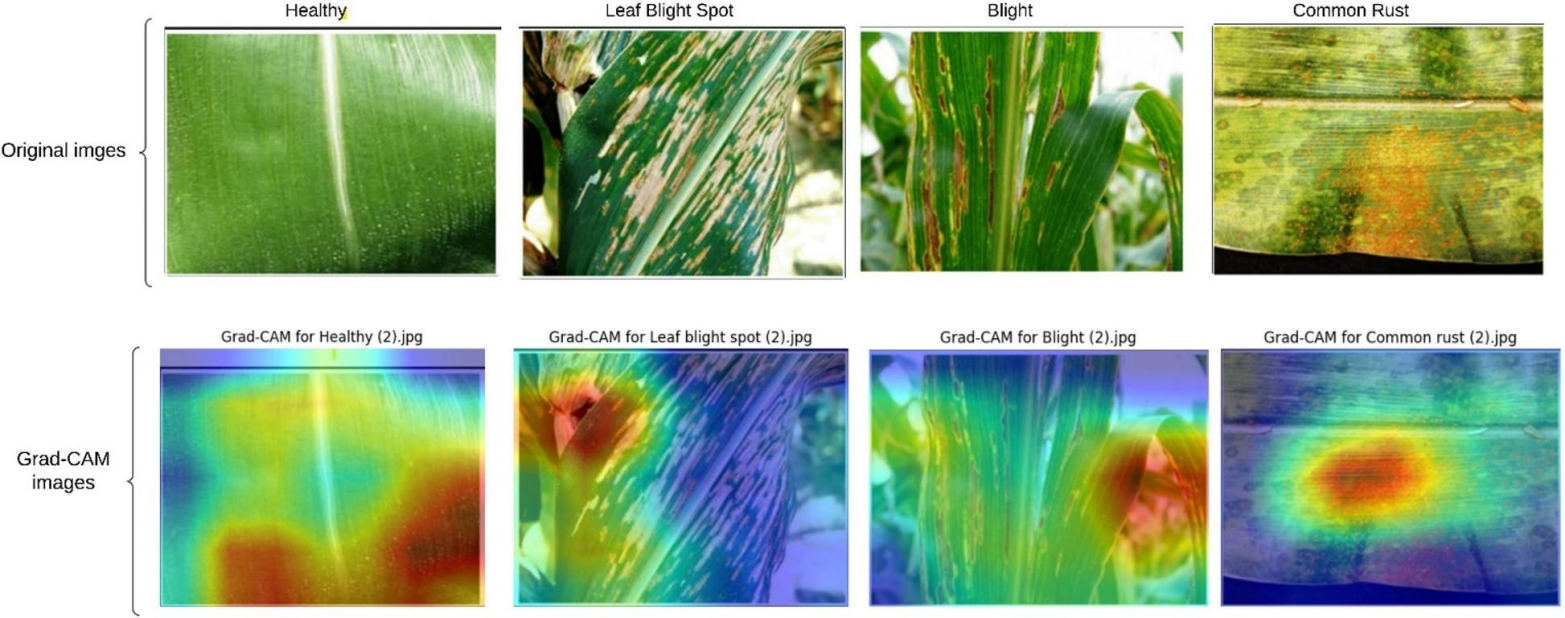
Peoposed Grad-CAM images for all four classes

Figure 10 illustrates the performance metrics of proposed model testing phase. To enhance the interpretability of our proposed ResNet152-based model for classifying corn leaf conditions, we incorporated Gradient-weighted Class Activation Mapping (Grad-CAM) shown in Fig. 11. By visualizing the regions of the input image that most influenced the model's predictions, Grad-CAM provides a layer of explainability critical for real-world applications. We applied Grad-CAM to the final convolutional layer (layer4[−1]) as it captures high-level semantic features. The generated heatmaps were resized to the original image dimensions and overlaid to provide interpretable visual evidence. Our analysis revealed that Grad-CAM consistently focused on disease-specific regions (e.g., blight lesions or rust spots), aligning well with expert knowledge. This capability not only validates the model's decisions but also fosters trust among stakeholders such as farmers and agricultural consultants. Future work will integrate quantitative evaluation metrics, such as Intersection over Union (IoU), to further validate Grad-CAM's reliability in agricultural disease detection.

The Fig. 11 showcases Grad-CAM visualizations for the classification of corn leaf conditions using ResNet152. The first row displays original images corresponding to four classes: Healthy, Leaf Blight Spot, Blight, and Common Rust. The second row presents Grad-CAM outputs for each image, highlighting regions that influenced the model's predictions. For healthy leaves, the Grad-CAM visualization shows diffuse activation, indicating uniform attention without specific disease-related focus. In contrast, for diseased leaves, the heatmaps concentrate on the affected regions, such as the blight lesions, rust-colored streaks, or blight spots, aligning well with visible disease symptoms. In order to improve the explainability of the proposed model, Grad-CAM visualizations were used to depict the classification decision-making regions of interest. The model correctly detected disease-specific patterns by concentrating on key regions of the leaf including lesions, discoloration, and rust spots. The Grad-CAM heatmaps for correctly classified samples suggest that the model focused on the relevant biologically important regions of the feature associated with each disease, which indicates sound feature extraction skills for the captured images.

In the misclassified cases, however, the heatmaps expose some relevant parts of the model's reasoning that appear to be flaws. For example, some misclassified images of leaf blight show attention spread out over the entire leaf rather than clumped in symptomatic areas. This suggests potential feature misinterpretation. Furthermore, some common rust misconceptions reveal the model active overhealthy and background veins, rather than disease symptomatic regions,behind actual disease symptoms. These findings reinforce the notion that the model captures disease features effectively in most cases, but does poorly distinguishing inter-class subtle differences, or visual similarities within the disease categories.Potential improvements consist of incorporating more advanced techniques of feature clarity, including noise reduction and contrast enhancement. Additionally, the model’s robustness may be improved by augmenting the dataset with more challenging samples for differentiating similar diseases. These features, which drive the model's accuracy, limit the model's understanding and, alongside Grad-CAM insights, help understanding shape the model's future classification accuracy improvements. This demonstrates the model's ability to localize and classify key disease features effectively, enhancing its interpretability and utility in real-world agricultural diagnostics.

### Discussion

Table 10 presents the metrics obtained by the model that classifies corn leaf diseases. The sensitivity of the model, or the percentage of each disease that has been correctly diagnosed, is almost excellent for all classes. For Blight, the sensitivity is found to be 99.78%, this is because the model is able to correctly diagnose this condition with an extremely low false negative rate of 0.22%. Common Rust is said to have a perfect score for sensitivity with a score of 100.0%; a high score, as there were no false negatives and the model was successful in identifying every example in this case. Likewise, Healthy plants' sensitivity is also 100%, signifying that all healthy leaf parts were correctly classified with no cases of misclassification. Gray Leaf Spot has a sensitivity of 99.76%, which was slightly lower than the other classes due to its 0.24% false negative rate. These overall results confirm the increased comprehensive ability of the model in the classification of corn leaf diseases with greater accuracy and significantly lesser errors. Based on the overall testing performance metrics, the accuracy of the model can be termed as astonishing, with virtually no errors in all classes.

**Table 10:** Overall metrics performance of proposed model training and testing phases

Classes	Se (%)	Acc (%)	Pr (%)	FNR (%)	Sp (%)	MR (%)
B	98.44	96.71	95.22	11.071	95.32	4.72
CR	99.58	98.98	98.34	3.02	99.35	1.48
GLS	92.03	96.31	80.12	9.95	97.11	4.82
H	99.76	99.94	99.69	0	99.84	0.06

**Algorithm 1** Corn Leaf Disease Classification Using ResNet152

Table 11 summarizes the performance metrics of the model for classifying corn leaf diseases into four categories: Blight, Common Rust, Gray Leaf Spot, and Healthy. Sensitivity (Se) measures the model's ability to correctly identify true positives, with Blight, Common Rust, Gray Leaf Spot, and Healthy achieving 98.44%, 99.58%, 92.03%, and 99.76%, respectively. Accuracy (Acc) reflects the overall correctness of predictions, ranging from 96.31% for Gray Leaf Spot to 99.94% for Healthy. Precision (Pr) evaluates how many of the predicted positives are correct, with values of 95.22% for Blight, 98.34% for Common Rust, 80.12% for Gray Leaf Spot, and 99.69% for Healthy. The False Negative Rate (FNR) highlights the percentage of actual positives missed, with lower values such as 3.02% for Common Rust and 0% for Healthy demonstrating strong performance. Specificity (Sp), which measures the ability to identify true negatives, ranges from 95.32% for Blight to 99.84% for Healthy. The Misclassification Rate (MR) is minimal across all classes, with Common Rust and Healthy having the lowest error rates of 1.48% and 0.06%, respectively. These metrics illustrate the robustness and reliability of the model in accurately identifying and categorizing corn leaf diseases.

**Table 11:** Overall metrics performance of proposed model training and testing phases

Metrics	Train	Test
Se (%)	99.88	97.45
Acc (%)	99.95	98.34
Pr (%)	99.88	98.02
FNR (%)	0.12	3.01
Sp (%)	99.96	97.91
MR (%)	0.05	1.12

Table 12, the performance metrics and training and testing phases of the model indicate its capability of classifying corn leaf diseases. At training stage, the model’s performance in terms of sensitivity (Se) was 99.88%, accuracy (Acc) was 99.95%, and precision (Pr) was 99.88% which shows a recognition of true positives and the associated prediction was very accurate. The false negative rate (FNR) at training stage was impressively low at 0.12% and the specificity (Sp) of the model was 99.96% which indicates that most of the true negatives were correctly classified. In addition, the research showed that the misclassification rate (MR) was very low, only 0.05%, indicating that the reliability of predictions is quite impressive.

**Table 12:** Accuracy comparison of proposed and other SOTA model

Author	Model	Accuracy (%)
[14]	MobileNetv3	98.26
[16]	EfficientNet	98.85
[17]	YOLOv5s-C3CBAM	83.00
[18]	YOLOv8	95.18
[19]	YOLOv5s (Lightweight)	98.45
[20]	DenseViT	98.85
[21]	MMF-Net	99.23
[22]	VIP-KNN	97.46
Proposed	ResNet152 with Grad-CAM (X-AI)	98.34
		99.95

In the testing stage, the model performance dropped slightly and maintained a sensitivity of 97.45% and an accuracy of 98.34%. The precision while testing the model reduced to 98.02% which was a decrease from the training phase but still a reasonable indication of the quality of predictions. During the tests, the false negative rate (FNR) to 3.01%, while the specificity was recorded at 97.91%. This indicates that the model was consistently preventing the misclassification of true negatives. The misclassification rate during the tests was 1.12% suggesting that the model is capable of performing well on new data while maintaining good classification accuracy. These findings support the inference that the model is able to perform well and generalise across many datasets, evidencing that it can learn quickly.

**Fig. 12:**
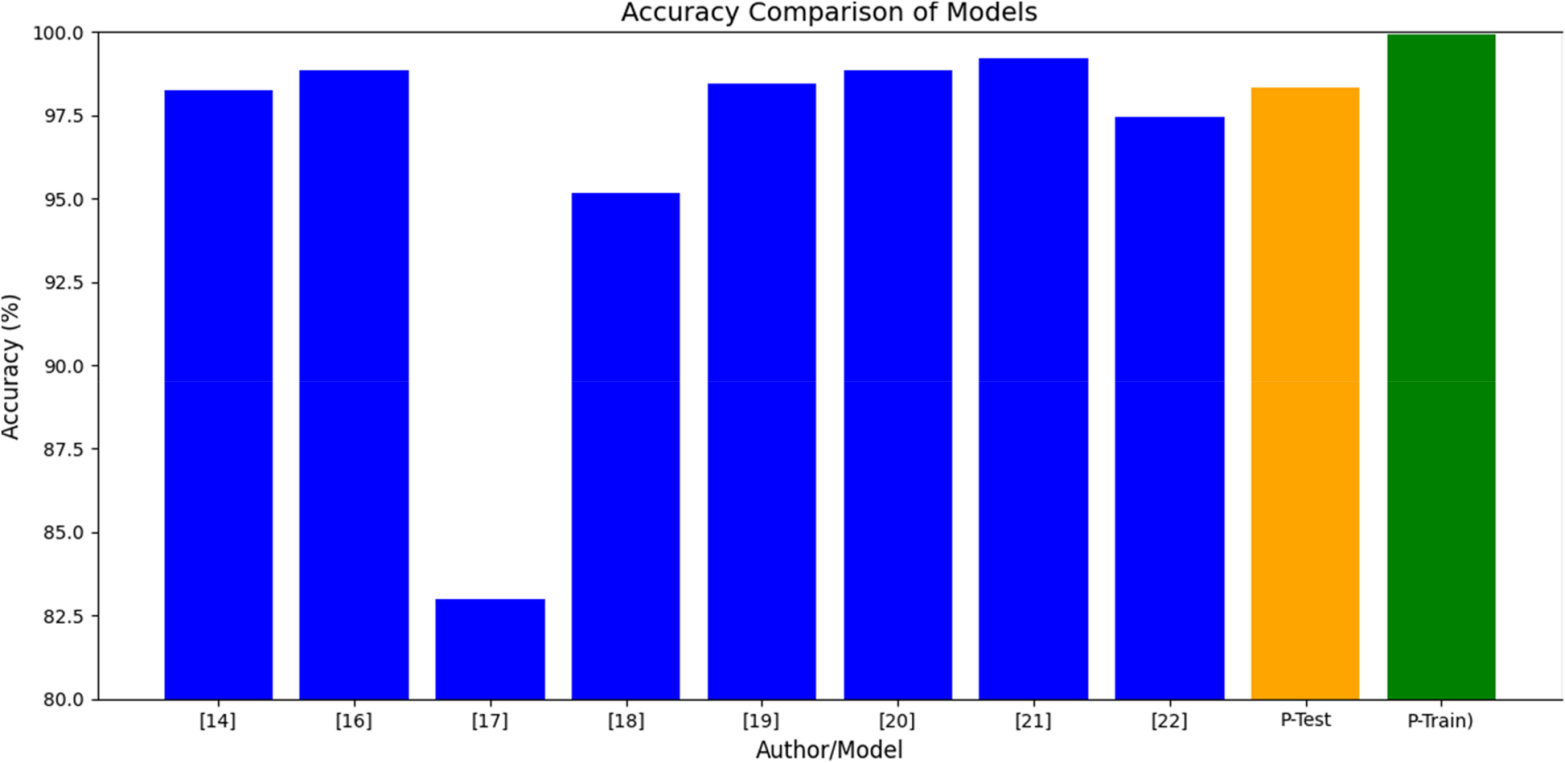
Proposed model accuracy comparison with other SOTA model

In terms of the results of model implementations, Table 11 presents a comparative efficacy in classification tasks of some other models. Out of the aforementioned models, MMF-Net was the most effective, managing to achieve an accuracy of 99.23%. Also, the ResNet152 with Grad-CAM (X-AI) presented good accuracy level of 98.34% and accuracy great peak of 99.95%, which means that the model is capable of performing complex tasks with high accuracy while still being explainable. Efficiency Net and DenseViT scored well and attained accuracy level of 98.85% while YOLOv5s (Lightweight) came close to them with an accuracy level of 98.45 percent. At 98.26 percent, MobileNetv3 recorded lower scores but remained highly competitive. In the case of the other models, YOLOv8 scored 95.18% and VIP-KNN made 97.46%. On the other hand, the results of YOLOv5s-C3CBAM were remarkably below average, providing only 83.00% of accuracy, which means it can be improved in some scenarios. These results point to the improvements that are seen in the model architectures while showing the performance cost and computational efficiency. Figure 12 depicts the accuracy comparison of proposed and SOTA models.

## Conclusion

In this study, the ResNet152 model is employed to process image data with the objective of detecting corn leaf diseases. It is clearly apparent from the results that the model outperformed the past works in all areas such as accuracy, specificity, misclassification and false positive rates. Thereby the model combined with integrated Grad-CAM with ResNet152 allows the farmers to understand the current condition of their corn crops due to the ability to diagnose the corn leaf diseases accurately. Such potential allows the farmers to strengthen the disease management operations enhancing the fruitful use of the resources while avoiding the big losses due to the high specific early diagnosis of the diseases. As a result, the model enables the farmers’ understanding of the disease cycle and disease control, crop systems, and other parameters of sustainable agriculture. This technology does not only support better decision making, but also demonstrates the value and importance of Explainable AI (X-AI) in smart agriculture because of the many measures of explanation and visuals it has. This study paves the way for future studies by demonstrating what explainable methods can do to improve the accuracy and trustworthiness of deep learning models in agricultural tasks.

The ResNet152 with Grad-CAM framework to recognize other types of crop’s diseases could be a promising direction for future work providing a wider context of this model. It also can leverage the IoT to provide modern real-time measures which relate with the early detection of crop diseases. There could also be an attempt to develop the model based on IoT applications on the active and passive mapping of the active models on weather phenomena and the quality of the soil. In addition, there are also methods of enhancing future understanding that focuses on enhanced visualization by means of more advanced explanatory techniques in order to support better decisions. Enabling mobile and edge computing systems may also enable farmers in dispersed regions to access the farming technologies. Additional studies could examine the feasibility of incorporating economic factors relevant to the adoption of such a technology by both small and large farmers. Creation of bias on sustainable developments such as enhancing disease management to avoid increased use of pesticides and improving methods on hybrid or ensemble models can have a huge positive impact. Planting such a system will benefit from plant pathologists, agronomists, and farmers and the whole structure might have a potential to improve agricultural approaches to more intelligent and green ones.

Our study demonstrates highly accurate classification corn leaf diseases, but agricultural field deployment in real-time is impeded by factors like site-specific computing resources, power availability, and rural internet access. Future work could look into aggressive sparsification strategies for mobile and edge devices to ensure useful application in farming contexts. Furthermore, the current model utilizes datasets specific to corn leaf diseases for both training and evaluation, which significantly restricts its applicability to other crop types. Other plant species can be included into this method but require domain adaptation approaches along with multi-crop dataset integration for increased model reliability. In addition, although the accuracy of the models is high, applying deep learning approaches in agriculture requires consideration of the technological know-how of the end users, the economics of and supporting infrastructure. Combining IoT-enabled supervision systems with mobile applications makes hassle-free interfacing possible, which might encourage adoption. The model predictions are also dependent on the datasets used for training them, creating bias if those datasets do not capture ample environmental conditions. Subsequent work should include datasets from different geographical locations and include climate, soil quality, and pest interactions as additional integrative factors for improved precision.

## Data Availability

The datasets used during the current study are available from the corresponding author on reasonable request. Data Availability Statement: The datasets used during the current study are available from the corresponding author on reasonable request.
